# Impact of annotation error in α-globin genes on molecular diagnosis

**DOI:** 10.1371/journal.pone.0185270

**Published:** 2017-10-19

**Authors:** J. Francis Borgio

**Affiliations:** Department of Genetic Research, Institute for Research and Medical Consultation (IRMC), Imam Abdulrahman Bin Faisal University (Formerly: University of Dammam), Dammam, Saudi Arabia; University of Naples Federico II, ITALY

## Abstract

**Background:**

Recent studies on the variants in duplicated human alpha globin genes (*HBA2* and *HBA1*) actively target the α-globin gene as molecular modulators for the treatment of β-thalassemia major. Identification of the exact position of variant in *HBA1*, *HBA2* or its patchworks is mandatory to support the therapeutic aims in β-thalassemia major, by identifying specific modulators for the reactivation of fetal hemoglobin production. Hence, accurate identification of the variants in α-globin genes is crucial for the proper diagnosis, treatment and genetic counseling.

**Method:**

The objective was to reveal the annotation errors produced in α-globin gene sequence analysis while using different analytic tools. An *HBA2* gene sequence with the HBA2:c.95+2_95+6delTGAGG variant and a recently reported *HBA12* gene convert have been taken as examples to prove annotation error in α-globin gene from different analytic tools.

**Results and discussion:**

Although various bioinformatics tools used to predict variants are usually of high reliability, the current study using the an alpha globin 2 sequence with the HBA2:c.95+2_95+6delTGAGG variant and a recently reported *HBA12* gene convert, has showcased ambiguous outputs among the three bioinformatics tools used and against the manual analytical method adopted.

**Conclusions:**

This report emphasizes the necessity for caution in the usage of DNA sequence analysis tools during molecular diagnosis and the importance of the selection of more appropriate tools for analysis. Furthermore, ethnic specific sequences should be considered as reference sequence for the analysis to bypass sequence dissimilarities among diverse populations.

## Introduction

Alpha globin genes are located in the *p* arm of chromosome 16. They are duplicated as *HBA2* (hemoglobin alpha 2) and *HBA1* (hemoglobin alpha 1), both the genes are highly homologous and encode 141 amino acid residues which make the alpha globin chain [1,2]. Normally, there are 4 alpha globin genes (α_2_α_1_/α_2_α_1_) in a healthy person. Almost 1000 globin gene variants have been reported from various populations [3,4]. Most researchers depend on web based free softwares or commercially available bioinformatics tools for the analysis of sequences to identify the variants. Precise identification of the DNA sequence variations in α-globin genes and its variants is mandatory for the proper diagnosis, treatment and effective genetic counseling to prevent progeny with Hb Bart’s hydrops fetalis syndrome. Furthermore, recent studies actively search for the actual part of the α-globin gene as a molecular target for the treatment of β-thalassemia [5]. This paper aims to reveal some of the dissimilarities in the analysis output of variants in α-globin genes when different bioinformatics tools were used.

## Materials and methods

An alpha globin 2 sequence with the HBA2:c.95+2_95+6delTGAGG variant and a recently reported *HBA12* [6] gene convert have been taken as examples to prove annotation error in α-globin gene from different analytic tools. The two sequences (HBA2:c.95+2_95+6delTGAGG and *HBA12*) with variants were given as input sequence and carefully analysed using Variobox v.1.4.6 [7], MAFFT version 7 (Multiple alignment program for amino acid or nucleotide sequences) [8] and Mutation Surveyor V4.0.8 [9]. Additionaly, Mutalyzer 2.0.22 was used to identify the gene conversion phenomenon [10,11]. Finally, all the results from the three tools were compared, the ambiguous results were manually checked. NG\_000006.1 was used as reference sequence.

## Result and discussion

**Fig 1:**
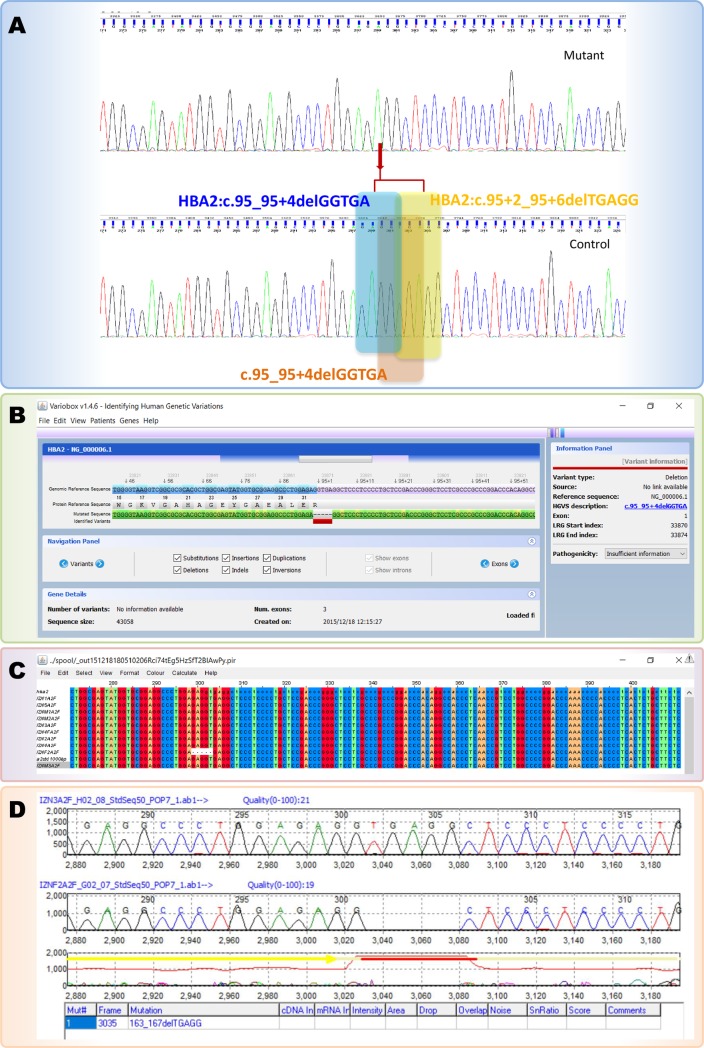
Output of the analysis of alpha globin 2 sequence with the HBA2:c.95+2_95+6delTGAGG variant using various tools. A: Electropherogram with the names from various tools. B: Sequence analysis results using Variobox v.1.4.6. C: Sequence analysis results using MAFFT version 7. D: Sequence analysis results using Mutation Surveyor V4.0.8.

The differences between the *HBA1* and *HBA2* genes have considered carefully for the analysis. An alpha globin 2 sequence with the HBA2:c.95+2_95+6delTGAGG or IVS I-1 (-5 bp) variant was analysed using various tools. The output of the analysis of the 5bp deletion (HBA2:c.95+2_95+6delTGAGG), which was reported already (HbVar ID 1065) [10,11] revealed three different names with various tools (Fig 1). The 5bp deletion (HBA2:c.95+2_95+6delTGAGG), was identified as HBA2:c.95_95+4delGGTGA using Variobox v.1.4.6 (Fig 1B). The analysis result from Variobox using the sequence (with HbVar ID 1065) appeared like a novel 5 bp deletion with the name HBA2:c.95_95+4delGGTGA. The same deletion (HbVar ID 1065) was identified as novel variant with the nomenclature, HBA2:c.93_95+2delGAGGT according to the MAFFT version 7 (Multiple alignment program for amino acid or nucleotide sequences) (Fig 1C). Furthermore, the same sequence (with HbVar ID 1065) was analysed using the Mutation Surveyor V4.0.8, with the variants 163_167delTGAGG, which appeared to be a novel (Fig 1D). The deletion of pentanucleotide (TGAGG) occurs within the exon1 and IVSI splice junction [12,13]. Different names such as HBA2:c.95_95+4delGGTGA, HBA2:c.93_95+2delGAGGT and 163_167delTGAGG by Variobox v.1.4.6, MAFFT version 7 and Mutation Surveyor V4.0.8 showed a clear contradiction on this deletion. In case of performing analysis with any one of these tool for the sequence analysis, author could strongly state that this variant as novel one. A deep manual cross checking and electropherogram comparison by the Mutation Surveyor V4.0.8 clearly describes that all these names are improper. HbVar is one of the best servers to cross check the presence of various variants before concluding novel variants (4), as on the HbVar have 348 and 431 different types of variants entries on the *HBA1* and *HBA2* genes respectively (http://globin.bx.psu.edu/).

**Fig 2:**
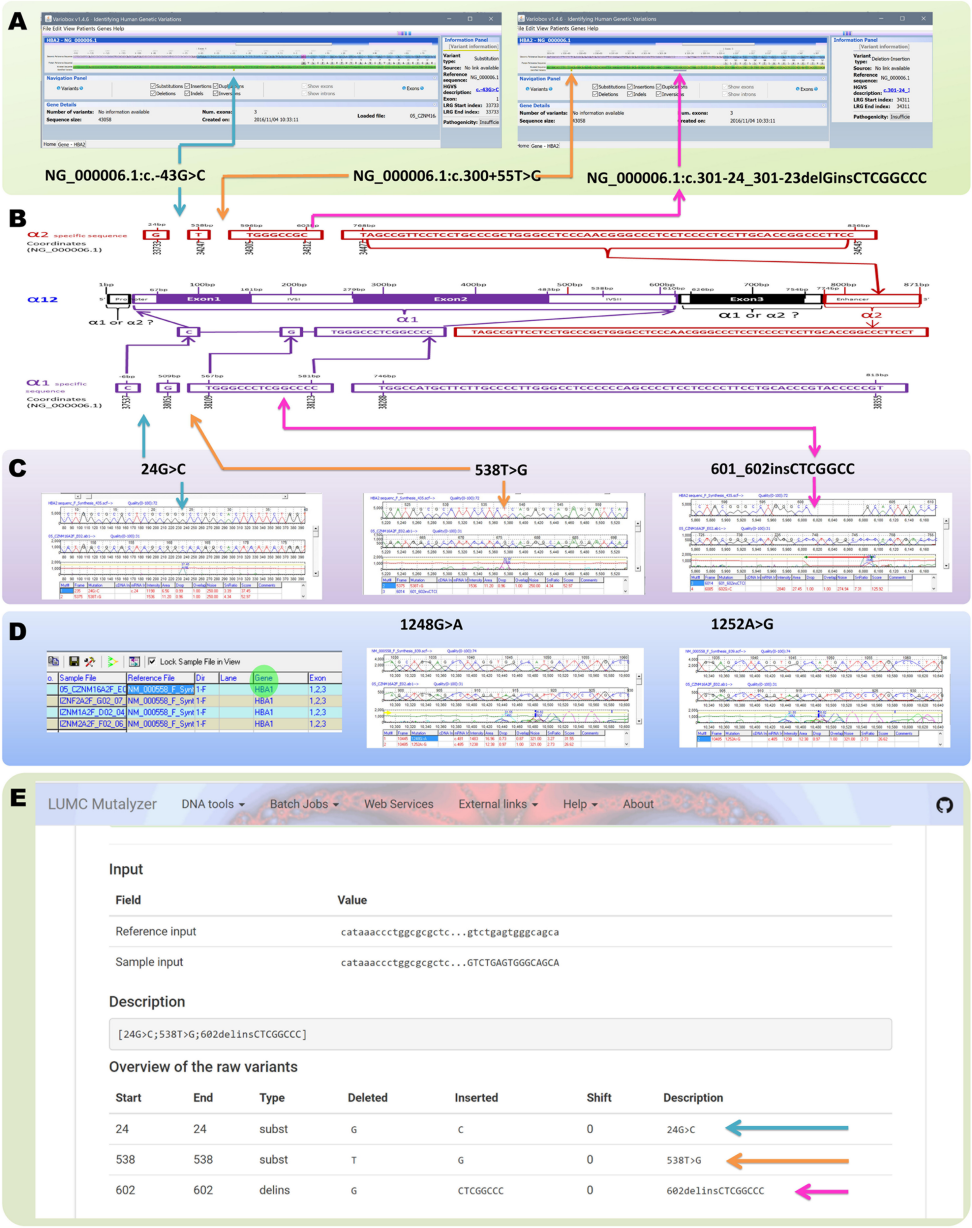
Alpha globin 12 gene analysis in different tools. A: Analysis results of the *HBA12* gene sequence using variobox. B: Structural elucidation of *HBA12* gene and the comparative similarities among the *HBA1*, *HBA2* and *HBA12* genes. C: Analysis results of the *HBA12* gene sequence using Mutation Surveyor V4.0.8 with reference gene (*HBA2*) input. D: Analysis results of the *HBA12* gene sequence using Mutation Surveyor V4.0.8 without reference sequence. The mutation surveyor fetched the reference sequence data from the database and displayed the *HBA2* sequence with part of *HBA1* sequence as *HBA1* gene (highlighted in green). E: Variant description extraction of the *HBA12* gene convert using Mutalyzer 2.0.22 with *HBA2* gene sequence as reference sequence.

The second example taken for the analysis is *HBA12* gene sequence [6], which was reported to be a combination of *HBA1* and *HBA2* gene sequences. Analysis using the web based free (Variobox v.1.4.6 and MAFFT version 7) and commercially available tool (Mutation Surveyor V4.0.8) failed to identify the gene conversion phenomenon. Instead all the tools displayed different variants as shown in the figure (Fig 2). Interestingly two different options during the analysis using the Mutation Surveyor resulted two different types of variants list on the *HBA12* gene convert. The first option with the input reference (*HBA2*) sequence resulted in two point substitution variant and an insertion (Fig 2C). The later one looked like a novel variant. The second option with auto fetching of the reference sequence based on the sample sequence, the Mutation Surveyor identified the *HBA2* gene convert sequence as *HBA1* gene (Fig 2D). The freely available software Mutalyzer was used to verify the variants and name them according to HGVS nomenclature rules [11]. *HBA2* gene sequence was considered as reference sequence and the *HBA12* gene sequence was used as sample sequence in variant description extractor tool at the Mutalyzer [10]. The Mutalyzer software could not to identify the gene conversion phenomenon (Fig 2E). The Mutalyzer software corrects HGVS nomenclature even for variants that have been incorrectly annotated [11]. However, the Mutalyzer did not fulfil the requirement of identifying the gene conversion phenomenon between the homologous genes. These results make the analysis even more complicated. If a researcher depends only on the analysis tools (online or commercial), he might end up in reporting “naturally not existing novel variants” in α-globin genes. Guidelines for variant nomenclature (http://varnomen.hgvs.org/) should be considered carefully before finalizing any novel variants.

There were 8 entries in the HbVar on the alpha thalassemia under the classification of “alpha (1 or 2 unclear) thalassemia” (http://globin.bx.psu.edu/). Probably these variants would have been determined at protein level and not verified at DNA level. These results should be reconsidered by the researchers for the proper classification of the variant in α-globin genes. Analytic tools and HbVar database should updated for the gene conversions reported between the *HBA1* and *HBA2* genes [6,14,15,16].

Ambiguous results were obtained while analyzing the sequences using three different bioinformatics tools as well as by manual cross validation and also careful literature review provide strong evidence that DNA sequence analytic tools may exhibit incorrect molecular diagnosis. Though variants could be almost accurately analyzed manually, it is unfeasible when the variant spectrum is wide and samples size is high. Therefore molecular biologists as increasingly rely on bioinformatics tools to identify variants. This paper gives an insight for the readers especially for the early career researchers to enhance the accuracy of the *HBA1* and *HBA2* sequence analysis. Identification of the position of variant in *HBA1*, *HBA2* or patchworks is mandatory to fulfill the therapeutic aims in β-thalassemia major, activation or the deactivation of specific alpha globin, identification of allele specific modulators to modulate the level of HbF (fetal haemoglobin). Annotation error should be avoided to enhance the up and down regulation of α-globin gene with α^+^ or α^0^ variants and to specify the malfunctioning alpha globin. Based on the breadth of the present observations, we can expect annotation errors in the next generation sequencing (NGS) data analysis, especially on the analysis of gene converts and mutations in the globin gene converts. Hence, curation methodologies are needed to reduce the NGS data annotation errors in the identification of mutations in the genes, which are prone to homologous recombination. Software and data bases designed to feed additional inputs such as standard controls sequences, ethnic control sequence from respective population, inheritance pattern would significantly reduce the annotation errors. Furthermore, ethnic specific sequences should be considered as reference sequence for the analysis to bypass sequence dissimilarities among diverse populations. This is the high time for the proper design of analysis software to identify the alpha globin gene variations with fewer miscalling, which could also be designed even more suitable for molecular diagnosis to be ideally validated.
